# The first reported case of Beaulieu-Boycott-Innes syndrome caused by two novel mutations in *THOC6* gene in a Chinese infant

**DOI:** 10.1097/MD.0000000000019751

**Published:** 2020-04-10

**Authors:** Qiang Zhang, Shaoke Chen, Zailong Qin, Haiyang Zheng, Xin Fan

**Affiliations:** a Laboratory of Genetic and Metabolism, Department of Paediatric Endocrine and Metabolism, Maternal and Child Health Hospital of Guangxi; b Department of Pediatrics, The Second Affiliated Hospital of Guangxi Medical University, Nanning, China.

**Keywords:** Beaulieu-Boycott-Innes syndrome, developmental delay-microcephaly-facial dimorphism syndrome, mutation, THOC6

## Abstract

**Rationale:**

This case report expands the mutation and phenotypic spectra of Beaulieu-Boycott-Innes syndrome (BBIS), and will be valuable for mutation-based pre- and post-natal screening of BBIS when conducting a genetic diagnosis.

**Patient concerns:**

A 4-year old boy from Guilin City, Guangxi Zhuang Autonomous Region, China, was referred to our clinic for clarification of his diagnosis because he showed moderate intellectual disability.

**Diagnosis:**

Two novel compound heterozygous mutations of THOC6, c.664T>C (p.Trp222Arg) and c.945+1 G>A were identified in this patient by whole exome sequencing. The two mutations were evaluated as pathogenic and likely pathogenic respectively according to the American College of Medical Genetics guidelines. This is the first case displaying the BBIS phenotype reported in the Chinese population. These two mutations have not been reported previously.

**Interventions:**

Symptomatic treatment and rehabilitation training for patients.

**Outcomes:**

The genetic cause of the disease was identified. The family received scientific genetic counseling.

**Lessons:**

BBIS is a rare syndromic autosomal recessive disease with intellectual disability and it is normally difficult for clinicians to recognize it. Whole exome sequencing is an efficient way to identify the gene which causes a particular disease in patients.

## 1 Introduction

Beaulieu-Boycott-Innes syndrome (BBIS) is a rare autosomal recessive neurodevelopmental disorder associated with the THO complex 6 gene (*THOC6*),^[1]^ and is clinically characterized by developmental delay, moderate to severe intellectual disability (ID) and subtle dysmorphic facial features. Other anomalies include microcephaly, cardiac and renal defects as well as cryptorchidism in males.^[1]^ THOC6 is part of the THO/TREX (transcription/export) complex, which is involved in mRNA transcription, processing and export of spliced mRNA.^[2]^ THO consists of THOC1, 2, 5, 6, and 7 as well as additional proteins. Complete knockouts of THOC1 and THOC5 were found to be lethal.^3^,^4^ Homozygous mutations and disruption caused by translocation can lead to ID, congenital ataxia, and cerebellar hypoplasia.^[5]^ So far, variants in the *THOC6* gene have been identified to associate with BBIS.

Here, we report on the first case of BBIS diagnosed by whole exome sequencing (WES) in a Chinese infant and neither of the mutations of BBIS described have not been published previously. This information will help to expand the mutation and phenotypic spectra of BBIS.

## 2 Case report

Ethical approval was obtained from the Ethics Committee of the Maternal and Child Health Hospital of Guangxi. A written informed consent was obtained from the parents. The proband was a 4-year old boy from Guilin City, Guangxi Zhuang Autonomous Region of China. He was referred to our clinic because he suffered from mental retardation. The boy was the first-born child of healthy and non-consanguineous parents at 37^+6^ weeks gestation. The birth was breeched and the infant had a birth weight of 2.66 kg and height of 50 cm. There was no history of asphyxia during the neonatal period, but the Apgar scores were not available. The boy was breastfed after birth, with feeding difficulty and weak sucking and dysphagia were observed. At the same time, he had recurrent respiratory infections during infancy.

**Figure 1:**
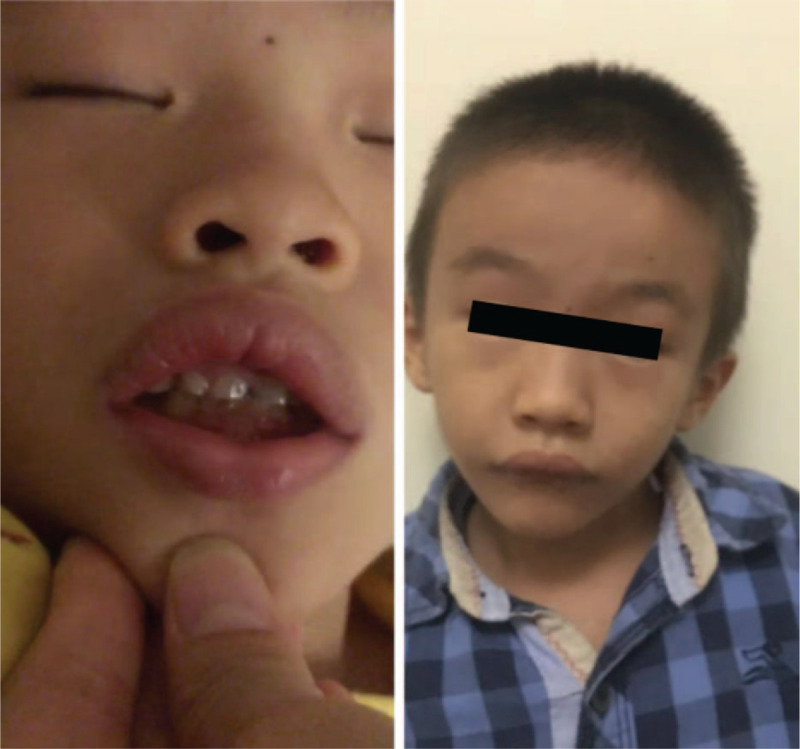
The facial features of the patient. A triangular face with a long jaw, long nose, protruding ears, an adducent lower lip, and the upper lip is thick and lifted.

Physical examination: the patient's height, weight, and head circumference were 93 cm (−3SD), 11.5 kg (−3SD), and 48 cm (−2SD), respectively. He had dysmorphic facial features (Fig. 1), including a triangular face, long jaw, long nose, high palate, and protruding ears as well as an adducent lower lip and the upper lip was thick and lifted. The muscle tone, hearing, and vision were normal.

Ultrasonic examination: ultrasound examination revealed the kidney, liver, and genital system were normal. Echocardiographic indicated a ventricular septal defect, atrial septal defect, and mesenteric cyst (post-operative). Magnetic resonance imaging (MRI) of the brain performed in the neonatal period was normal and MRI scans of the brain cannot be repeated because of his previous cardiac surgery.

Laboratory examination: Some biochemical tests, metabolic tests (bloodspot amino acids and acyl carnitines/urine organic acids test), and chromosomal microarrays were performed. WES was, then, performed for which genomic DNA samples were captured to create sequencing libraries using an Agilent Sure Select Human All Exon V5 Kit (Agilent Technologies, Santa Clara, CA) in accordance with the manufacturer's protocol. The prepared libraries were sequenced with a HiSeq2500 system (Illumina, San Diego, CA).

The results of biochemical and metabolic tests as well as chromosome analysis were normal, but *THOC6* gene compound heterozygosis variations c.664T>C (p.Trp222Arg) c.945+1 G>A (NM_02 4339.3, Chr 16:3077039, and Chr 16: 3077502) were found by WES. Sanger sequencing was used to identify the mutations following PCR amplification using primers: 5’GAGGCCCTGTGTCTCACTTC3’and 5’CCAGGTTGGTGAAGACATCC3’ for c.945+1G>A/and 5’GTCCTCTTCTCCCCCAACTC3’ and 5’TGGACAGAAAGGTGGGAGTC3’ for c.664T>C/p.Trp222Arg.

**Figure 2:**
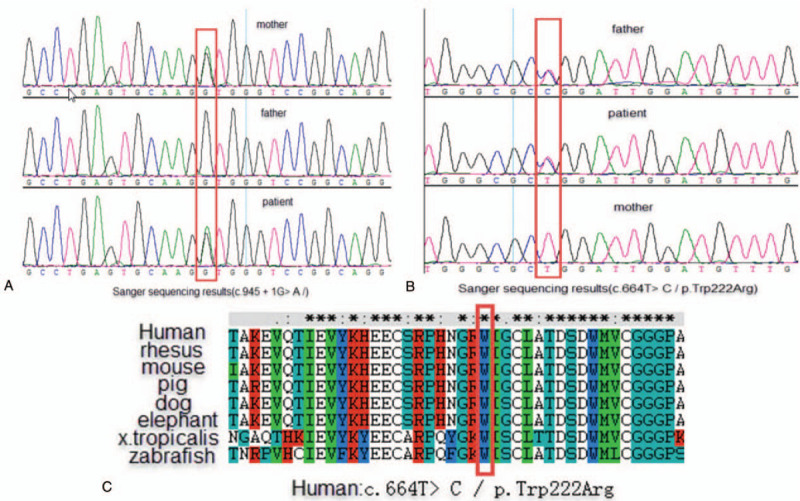
(A) and (B) are sequencing figures of the compound heterozygous mutations and (C) is the conservative analysis figure of c.664 T>C (p.Trp222Arg).

**Figure 3:**
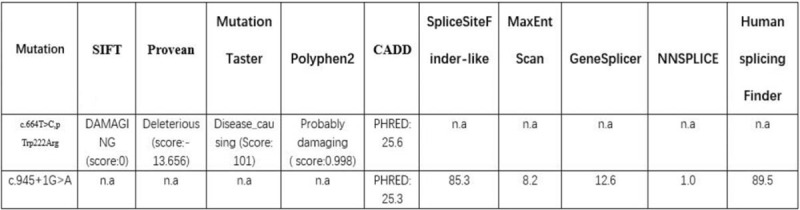
In silico predictions. The impact of each of the THOC6 variants was predicted using five in silico tools.

Sanger validation indicated that the c.664T>C (p.Trp222Arg) mutation was inherited from father, while the c.945+1 G>A mutation was inherited from the mother (Fig. 2A and B). The two variants were absent from controls, including the local population database and the gnomAD (http://gnomad.broadinstitute.org). In contrast to the weak effects of common SNPs, rare single nucleotide variants would have highly penetrant and deleterious effects on the phenotype.^[6]^ We predicted the impact of c.664T>C/p Trp222Arg with five in silico tools: SIFT, Provean, Mutation Taster, Polyphen2, and CADD (Fig. 3). At the same time, CLUSTAL V was used for conservative analysis of this mutation (Fig. 2C). Predictive software suggested that c.664T>C/p Trp222Arg was a harmful mutation, and sequence homology analysis revealed that it was conservative. The mutation of c.664T>C/p Trp222Arg was assessed to be likely pathogenic (PM1, PM2, PM3, PP2, and PP4) by the ACMG/AMP guidelines.

According to the recommendation of UV (unclassified variants) guidelines for splice mutation analysis, three prediction algorithms should be used in order to have a consensus prediction.^[7]^ So, the impact of c.945+1G>A was predicted by ALAMUT VISUAL (https://www.interactive-biosoftware.com/alamut-visual/), the software includes a splicing module, integrating a number of prediction algorithms and splicing prediction data (SpliceSiteFinder-like, MaxEntScan, GeneSplicer, NNSPLICE, and Human Splicing Finder). The prediction software prompted the c.945+1G>A was a donor site mutation which was most probably affected by splicing (Fig. 3). When it is mutated, the splicing pattern of the pre-mRNA will change. Therefore, the c.945+1G>A mutation was assessed as pathogenic by the ACMG/AMP 2015 guidelines (PVS1, PM2, and PM3). In addition, the two mutations mentioned above would most likely both cause serious defects in gene function. As generally believed, loss of function (LOF) is the pathogenic mechanism of recessive genetic disease and we believe these two mutations would be the disease causing mutations in a patient.

**Figure 4:**
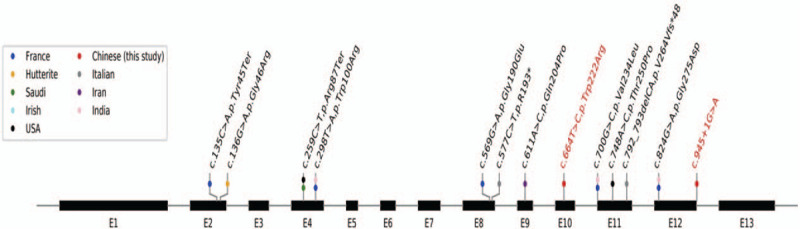
Possible mutations in *THOC6* gene.

A literature review on the different genotypes and phenotypes found in BBIS patients was performed. To date, 14 patients with 11 different THOC6 mutations have been reported (Fig. 4). The associations of the patient's phenotype and genotype are shown in Table 1.

**Table 1:**
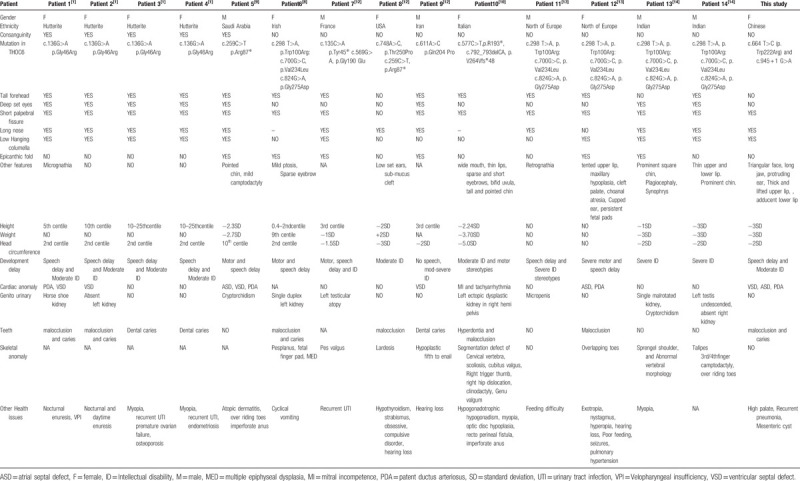
The patient's phenotype and genotype association.

## 3 Discussion

BBIS is a genetic syndrome, with core clinical features including ID with language delay, facial dysmorphism and congenital renal, and cardiac malformations.^1^,^8^,^9^ A new report indicated additional features included severe vermian dysgenesis and hydrocephalus due to aqueductal stenosis, multiple skeletal anomalies, and hyper-gonadotropic hypogonadism.^[10]^

In this study, the patient showed many of these clinical features, such as mental retardation, especially language development delay, short stature, subtle abnormal facial features, and cardiac abnormalities including VSD, ASD, and PDA. Previous studies on BBIS are summarized in Table 1 and facial features were frequently observed among patients with mutations in THOC6, including a tall forehead (12/16), short- and up-slanted palpebral fissures (14/16)/deep set eyes (8/16), a long nose (12/16), and low-hanging columella (10/16). The clinical features of microcephaly, weight loss, malocclusion, and caries were also very common in cases with BBIS syndrome. Our patient presented with similar as well as with different facial features, included a triangular face, thick upper lip vermilion, lower lip adduction, and retrognathia. Most of the facial features of patients with BBIS syndrome were non-specific, or were even different between different ethnic groups, so the clinical diagnosis of BBIS syndrome can be very challenging to clinicians. Additionally, the patient in this study presented with several non-specific clinical manifestations including feeding difficulties, mesenteric cysts, recurrent pneumonia and a high palate which subsequently extended the clinical manifestations of this disease.

The *THOC6* gene is a component of the THO complex and it interacts with additional components to form the TREX complex (transcription export complex) which seems to have a dynamic structure involving ATP-dependent remodeling.^2^,^7^ The TREX complex plays an important role in the apoptotic negative control involved in the development of the brain.^[1]^ *THOC6* is located at 16p13.3 region of the chromosomes (chr16:3,024,027–3,027,755, GRCh38/hg38). It is composed of 3729 bases which translates into 341 amino acids and it is mainly localized in nuclear speckles and nucleoplasm 12.^[11]^ Mutations in THOC6 have been identified in different populations worldwide and it has been validated as a disease causing gene of BBIS syndrome.

So far, 11 mutations in THOC6 have been reported (Fig. 4) and most of which were missense ones. In this study, two novel variants c.664T>C/p. Trp222Arg and c.945+1G>A were reported and three different software packages were used to predict the impact of these mutations. The prediction indicated the two variants were both potentially pathogenic and functional studies are needed to prove the pathogenicity of these mutations.

BBIS with non-specific features is difficult to be recognized by clinicians. The presentation of ID along with subtle characteristic facial features and various dimorphisms should provide a diagnostic clue for the presence of BBIS. WES is an efficient way to find the disease causing gene of these patients. As is reported in the literature, most of the verified BBIS patients were also diagnosed by clinical features and WES or WES alone.

## 4 Conclusions

This study identifies two novel compound heterozygous variants of the *THOC6* gene in a Chinese patient, who expressed ID, subtle facial features, short stature, cardiac abnormality, recurrent pneumonia, and mesenteric cysts. The mutations and clinical symptoms reported in this study enrich the BBIS mutation spectrum and extend the phenotype spectrum of the disease in different ethnic groups and this may prove valuable for future mutation-based screening and genetic diagnosis.

## Acknowledgments

We would like to thank the family members of our patient for their assistance with the clinical evaluation. The authors would like to thank Dr. Dev Sooranna, Imperial College London, for editing the manuscript.

## Author contributions

All authors read and approved the final manuscript.

**Conceptualization:** Qiang Zhang.

**Data curation:** Qiang Zhang.

**Formal analysis:** Qiang Zhang.

**Methodology:** Qiang Zhang.

**Resources:** Qiang Zhang.

**Software:** Qiang Zhang.

**Writing – original draft:** Qiang Zhang.

Qiang Zhang: 0000-0001-6203-0967.
